# Hydrocephalus due to multiple ependymal malformations is caused by mutations in the *MPDZ* gene

**DOI:** 10.1186/s40478-017-0438-4

**Published:** 2017-05-01

**Authors:** Pascale Saugier-Veber, Florent Marguet, François Lecoquierre, Homa Adle-Biassette, Fabien Guimiot, Sara Cipriani, Sophie Patrier, Marie Brasseur-Daudruy, Alice Goldenberg, Valérie Layet, Yline Capri, Marion Gérard, Thierry Frébourg, Annie Laquerrière

**Affiliations:** 1 Department of Genetics, Normandie Univ, UNIROUEN, INSERM U1245, Normandy Centre for Genomic and Personalized Medicine, Rouen University Hospital, F76000 Rouen, France; 2 Department of Pathology, Normandie Univ, UNIROUEN, INSERM U1245, Rouen University Hospital, F76000 Rouen, France; 3 Department of Pathology, Lariboisière Hospital, APHP, F75000, Paris Diderot University, Sorbonne Paris Cité, PROTECT INSERM, F75019 Paris, France; 4 Department of Genetics and Cytogenetics, Foetopathology Unit, Robert Debré Hospital, APHP, Paris Diderot University, INSERM U1141, F75019 Paris, France; 5 Paris Diderot University, Sorbonne Paris Cité, PROTECT INSERM, F75019 Paris, France; 6 Department of Pathology, Rouen University Hospital, F76000 Rouen, France; 7 Department of Radiology, Rouen University Hospital, F76000 Rouen, France; 8 Department of Genetics, Normandy Centre for Genomic and Personalized Medicine, Rouen University Hospital, F76000 Rouen, France; 9 Department of Genetics and Cytogenetics, Le Havre Hospital, F76600 Le Havre, France; 10 Department of Genetics, Robert Debré Hospital, APHP, F75019 Paris, France; 11 Department of Genetics, Normandy Centre for Genomic and Personalized Medicine, Caen University Hospital, F14000 Caen, France; 12 Pathology Laboratory, Pavillon Jacques Delarue, CHR,1 rue de Germont, 76031 Rouen Cedex, France

**Keywords:** *MPDZ* pathogenic variants, Foetal hydrocephalus, Neuropathology, Multifocal malformation of the ependyma, Autosomal recessive inheritance

## Abstract

Congenital hydrocephalus is considered as either acquired due to haemorrhage, infection or neoplasia or as of developmental nature and is divided into two subgroups, communicating and obstructive. Congenital hydrocephalus is either syndromic or non-syndromic, and in the latter no cause is found in more than half of the patients. In patients with isolated hydrocephalus, *L1CAM* mutations represent the most common aetiology. More recently, a founder mutation has also been reported in the *MPDZ* gene in foetuses presenting massive hydrocephalus, but the neuropathology remains unknown. We describe here three novel homozygous null mutations in the *MPDZ* gene in foetuses whose post-mortem examination has revealed a homogeneous phenotype characterized by multiple ependymal malformations along the aqueduct of Sylvius, the third and fourth ventricles as well as the central canal of the medulla, consisting in multifocal rosettes with immature cell accumulation in the vicinity of ependymal lining early detached from the ventricular zone. MPDZ also named MUPP1 is an essential component of tight junctions which are expressed from early brain development in the choroid plexuses and ependyma. Alterations in the formation of tight junctions within the ependyma very likely account for the lesions observed and highlight for the first time that primary multifocal ependymal malformations of the ventricular system is genetically determined in humans. Therefore, *MPDZ* sequencing should be performed when neuropathological examination reveals multifocal ependymal rosette formation within the aqueduct of Sylvius, of the third and fourth ventricles and of the central canal of the medulla.

## Introduction

Hydrocephalus which literally means any increase in cerebrospinal fluid (CSF) within the skull has been more precisely defined by the International Hydrocephalus Working Group which describes “an active distension of the ventricular system resulting from inadequate passage of cerebrospinal fluid from its point of production within the cerebral ventricles to its point of absorption into the systemic circulation” [24]. In infants, its prevalence varies between 1 and 32 per 10.000 births and has been estimated by Munch et al. at 1.1 per 1,000 infants when including cases diagnosed before 1 year of age in the absence of other extrinsic causes and after exclusion of neural tube defects [14]. Several classifications have been proposed depending on the pathophysiological mechanisms, aetiology, or treatment modalities. In the aetiological classification proposed by Tully and Dobyns [24], congenital hydrocephalus is considered either as acquired representing about half of the cases and mainly due to haemorrhage, infection or neoplasia, or of developmental nature, also termed “intrinsic hydrocephalus”. This pathological condition is also separated into two subgroups, i.e., communicating i.e., with no point of obstruction or resistance to cerebro-spinal fluid dynamics, or obstructive, knowing that most of the time obstruction is the major cause of hydrocephalus. As regards obstructive hydrocephalus physiopathology, multiple points of obstruction have been recognized, including foramina of Monroe, aqueduct of Sylvius, 4^th^ ventricle foramina, spinal and cortical subarachnoid spaces, and from birth arachnoid villi and venous hypertension [17]. Moreover, several points of obstruction may coexist in a same patient. Regarding developmental causes, hydrocephalus is classically divided into syndromic (representing about 75% of the cases and due to chromosomal abnormalities in 30% of them, thus accounting for 6% of all causes of hydrocephalus), and non-syndromic forms. But the distinction between them is most of the time difficult, since additional anomalies may be present in apparently non-syndromic forms. No specific cause is found in more than half of the patients although they present a syndromic form in 11% of the cases, with only 0.6% of their whole infantile series having an identifiable genetic syndrome [24]. In individuals with apparently isolated hydrocephalus or with no major additional clinical findings, *L1CAM* mutations represent the most common genetic form with a prevalence of approximately 1:30,000 and account for about 5–10% of males with non-syndromic congenital hydrocephalus [19]. *L1CAM* pathogenic variants are responsible for a wide spectrum of phenotypes, now termed L1, the most severe form being Hydrocephalus with Stenosis of the Aqueduct of Sylvius (HSAS; MIM#307000). More than 200 different deleterious variants spanning over the entire gene have been reported so far [21, 28, 30]. In HSAS, the stenosis of the aqueduct of Sylvius is a hallmark of the disease together with hydrocephalus, adducted thumbs, pyramidal tract agenesis/hypoplasia, corpus callosum agenesis/hypoplasia and cerebellar anomalies [1, 27]. More recently, mutations in other genes have been reported to be causative for non-syndromic hydrocephalus, notably in the *MPDZ* gene (MIM#615219)*.* In 2013, a founder mutation in this gene was identified in two consanguineous Saudi families in whom the foetuses presented ultrasonographically massive bilateral hydrocephalus [2], but no post-mortem examination could be obtained, so that the underlying mechanism of hydrocephalus remained unknown.

In a previous work, we reviewed the neuropathology of 138 cases genetically tested for X- linked hydrocephalus [1] that allowed us to classify patients who did not display any pathogenic variant in the *L1CAM* gene into four distinct subgroups. Among them, atresia/forking of the aqueduct of Sylvius represented 27% of the cases, associated in some foetuses with rhombencephalosynapsis, fusion of the colliculi, atresia of the 3^rd^ ventricle, corpus callosum abnormalities consisting of partial/complete total agenesis or hypoplasia, and pyramidal tract hypoplasia or asymmetry. Though atresia of the aqueduct of Sylvius is usually considered as resulting from haemorrhage or infection, an autosomal recessive mode of inheritance was suspected in two consanguineous families, one in whom a child died from severe hydrocephalus and a foetus was interrupted for recurrent hydrocephalus, and another family in whom three foetuses were affected. Since then, recurrent massive hydrocephalus leading to the termination of the pregnancy occurred in an additional consanguineous family. We describe three novel homozygous *MPDZ* null mutations in these three families along with the prenatal phenotype and foetal neuropathological lesions, and provide some insights into the potential pathological mechanisms.

## Materials And Methods

### Patients

**Fig. 1:**
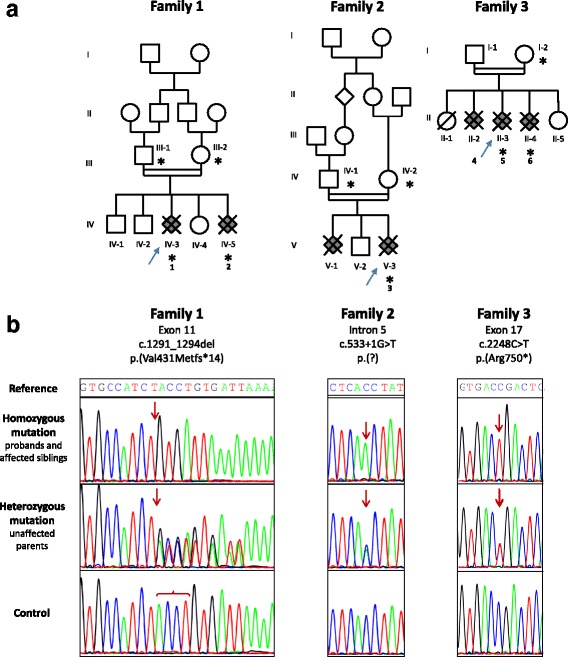
Identification of *three* homozygous mutations in the *MPDZ* gene using targeted NGS. Pedigrees of the *three families* with individuals affected by congenital hydrocephalus represented in *grey, blue arrows* indicate the affected foetuses which underwent NGS screening. An *asterisk* depicts individuals who underwent Sanger sequencing for segregation analysis. *Figures* under the foetuses identify the foetuses included in the study (**a**) Sanger sequencing electropherograms obtained in probands, affected siblings and parents (**b**)

The pedigrees of the three families are depicted on Fig. 1.

#### Family 1

**Fig. 2:**
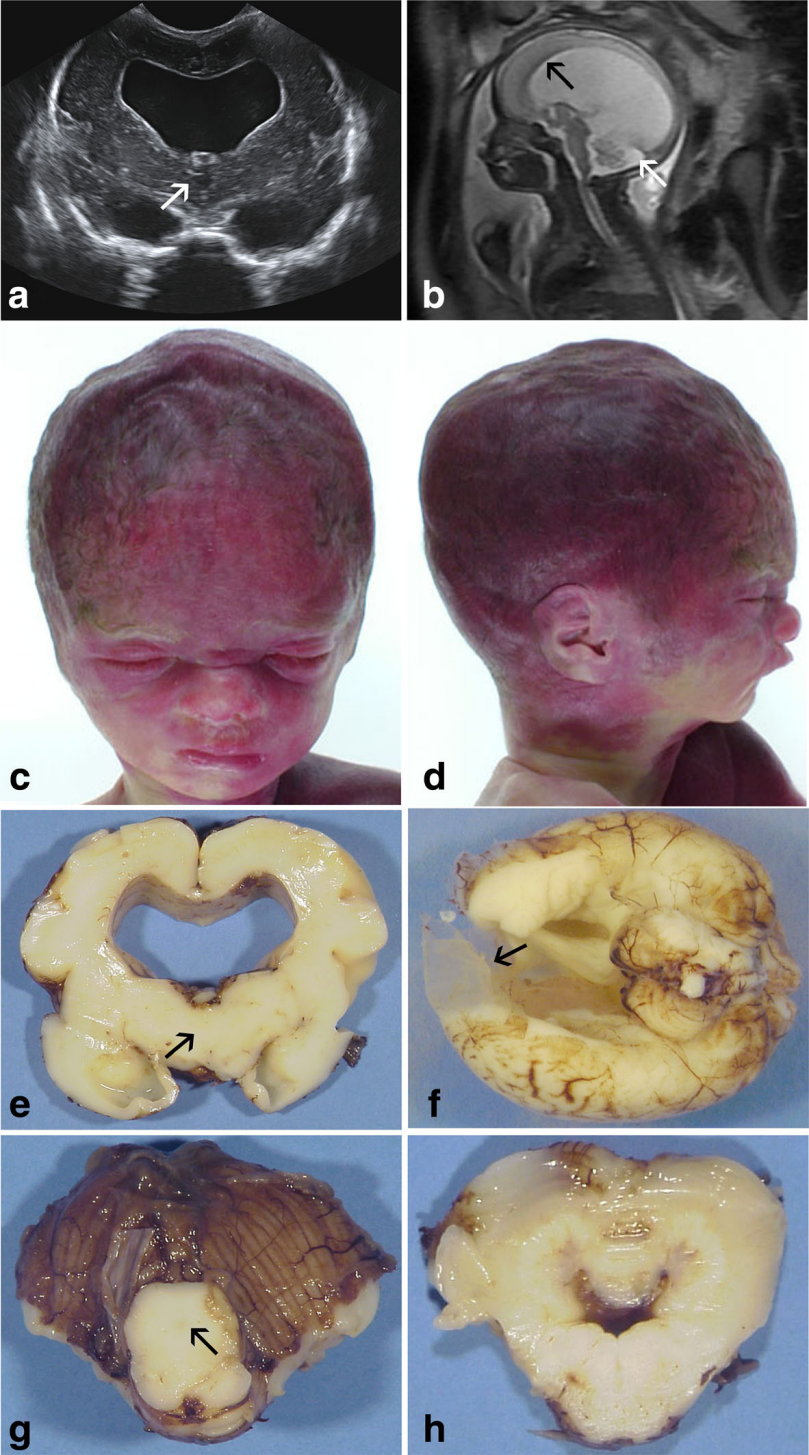
US coronal section passing through the diencephalon displaying absent third ventricle (*white arrow*) with major dilatation of the lateral ventricles and rupture of the septum in foetus 1 (**a**) and with on MRI considerable thinning of the corpus callosum in foetus 2 (*black arrow*), small vermis with enlarged cisterna magna (*white arrow*) (**b**). All foetuses presented characteristic dysmorphic features associating *prominent forehead, small nose* with *large nasal bridge and bulbous tip, small mouth* and *midface hypoplasia* (Foetus 4) (**c**) short prominent philtrum and micro-retrognathism (**d.**) On macroscopic section (Foetus 1), the corpus callosum was extremely thinned with undiscernible third ventricle (*arrow*) (**e**) and bilateral frontal brain parenchyma loss observed in foetus 2 due to severe intraventricular hyperpressure (*arrow*) (**f**) In the mesencephalon, the aqueduct was also undiscernible (*arrow*) (**g**) with in foetus 1 severe deformation of the fourth ventricle (**h**)

A 25-year-old woman, gravida IV, para II, underwent ultrasonography (US) at 22 weeks of gestation (WG), which revealed macrocephaly (head circumference > > 97^th^ percentile) with severe bilateral ventriculomegaly and rupture of the septum (Fig. 2a), but with no other associated brain, visceral or growth parameter abnormalities (Foetus 1). MRI performed at 28WG confirmed massive hydrocephalus (Fig. 2b). Based on these findings, a medical termination of the pregnancy was achieved at 30 WG in accordance with the French law. Chromosomal analysis performed on amniotic fluid cells revealed a normal female karyotype, 46, XX. Five years later, medical termination of the pregnancy was anew performed at 29 WG after the discovery of similar brain lesions on US (severe ventriculomegaly with anterior to posterior horns measured at 27 mm) in a female foetus (Foetus 2). Three children were previously born at term to these healthy Senegalese consanguineous parents of the first degree, and were also in good health.

#### Family 2

In this gravida III, para I 30-year-old woman, routine US performed at 21 WG revealed foetal macrosomia with severe isolated ventriculomegaly, that led to the termination of the pregnancy at 25 WG (Foetus 3). The karyotype was normal, 46, XY. A healthy male child was previously born at term, but a first female child, in whom foetal ventriculomegaly had been identified at 23 WG by US, was delivered at 35 WG. The neonate weighed 2920 g, head circumference was measured at 41.5 cm (> > 95^th^ percentile). Soon after birth, she underwent ventricular shunting, but died within a few weeks from meningoencephalitis. Autopsy was not performed. As in family 1, the parents were consanguineous of the first degree.

#### Family 3

In the third family, two female healthy children were born to consanguineous parents of the second degree with no particular familial medical history. Nevertheless, the mother underwent a termination of the pregnancy for renal anomalies in a female foetus, and three medical terminations of the pregnancies were achieved at 29 WG (Foetus 4), one year later at 25 WG (Foetus 5) and the following year at 23 WG (Foetus 6) for severe apparently isolated recurrent hydrocephalus. Since then, the mother had two other healthy children from a second marriage.

### Post-mortem examination

#### Autopsy procedures

A complete autopsy was performed in the five foetuses with the informed written consent of the parents in accordance with the French law and following standardized protocols. Foetal biometric data were evaluated according to Guilhard-Costa et al. [7].

#### Neuropathological evaluation

With the exception of family 3 in which only some systematic paraffin embedded blocks were taken from the brain and sent to our laboratory, foetal brains in family 1 and 2 of were fixed in a 10% formalin-zinc buffer solution for at least one month. Brain growth and macroscopic assessment of brain maturation including gyration were evaluated according to the criteria of Guihard-Costa and Larroche and the atlas of Feess-Higgins and Larroche respectively [6, 8]. Eight-micrometer sections obtained from paraffin-embedded tissues were stained using Haematoxylin-Eosin, and with Kluver Barrera in the third case (family 2). Immunohistochemical studies were performed on cases 1 and 3 using antibodies directed against vimentin (diluted 1:100; Dakopatts, Trappes, France), glial fibrillary acidic protein (GFAP, 1:300; Dakopatts), S100B protein (diluted 1:250, Dakopatts), epithelial membrane antigen (EMA, diluted 1:100, Dakopatts) pan-cytokeratin AE1/AE3 (diluted 1:100, Dakopatts), CD56 (N-CAM, diluted 1:100, Genemed Biotechnologies, San Francisco, USA), nestin (rabbit polyclonal, diluted 1:100, Millipore, Molsheim, France) and SOX2 (diluted 1:.100, Abcam, Paris, France). All immunolabelings were compared with three age-matched controls whose brain examination was completely normal. Immunohistochemical procedures included a microwave pre-treatment protocol to aid antigen retrieval (pretreatment CC1 kit, Ventana Medical Systems Inc, Tucson AZ). Incubations were performed for 32 minutes at room temperature using the Ventana Benchmark XT system. After incubation, slides were processed by the Ultraview Universal DAB detection kit (Ventana).

For confocal analyses, double immunolabelings were performed using multiple PDZ Domain protein antibody (MPDZ, diluted 1:400, Antibodies-online Gmbh, Aachen, Germany) and EMA (diluted 1:400, Dakopatts), as well as using nestin antibody (mouse monoclonal, diluted 1:500, Millipore) and PAX6 (rabbit polyclonal, diluted 1:100, Proteintech, Manchester, UK). Sections were incubated with primary antibodies overnight at 4 °C, and then treated with Alexa Fluor 488-, Alexa Fluor 555-, or Alexa Fluor 676-conjugated secondary antibodies (1: 500 in blocking solution, Invitrogen Molecular Probes) for 1 h at 25 °C. Nuclei were labelled with 4′, 6-diamidino- 2-phenylindole (DAPI, 1 μg/mL, Invitrogen Molecular Probes). Fluoromount-G mounting medium (Southern Biotech, Birmingham, USA) was used to mount coverslips. Confocal images were acquired using a CLSM Leica laser-scanning confocal microscope.

### Genetic analyses

#### DNA extraction

Genomic DNA was extracted from blood samples of foetus IV-3 in family 1 (Foetus 1) and her parents using QuickGene DNA Whole Blood Kit L (Kurabo, Japan), from blood samples of foetus V-3 in family 2 (Foetus 3) and his parents using QIAamp DNA Blood Midi Kit (Qiagen, Courtaboeuf, France), according to the manufacturer’s instructions, and from blood sample of foetus I-2 in family 3 using a manual salting-out extraction method. Genomic DNA was extracted from a post mortem biopsy tissue sample in foetus II-3 from family 3 (Foetus 5), from amniotic cells in foetus II-4 from family 3 (Foetus 6) and in foetus IV-5 from family 1 (Foetus 2) using a manual salting-out extraction method.

#### Targeted gene panel for non-syndromic hydrocephalus

A total of 11 genes were targeted for capture and deep sequencing. They include the 3 validated genes involved in non-syndromic hydrocephalus, *L1CAM*, *MPDZ* and *CCDC88C,* as well as eight candidate genes on the basis of mouse models of hydrocephalus (panel list available upon request). Using Sure Design (Agilent Technologies Inc., Santa Clara, California, USA), the capture was designed to include the whole *L1CAM* gene and all the coding exons from the 10 other genes with at least 50 bp of the flanking intronic sequence. In addition, 150 pb of the 5’UTR and 3’UTR regions were added to the targeted regions. NGS library preparations and target enrichment were performed with the SureSelect QXT kit (Agilent Technologies Inc., Santa Clara, California, USA): in-solution hybridization capture using custom biotinylated RNA baits was performed according to the manufacturer’s instructions. The multiplexed samples were sequenced on the Illumina Miseq platform (Illumina, San Diego, California, USA) using 150-bp paired-end reads.

#### Variant analysis and prioritization

Output paired-end sequencing data were processed through two independent bioinformatical pipelines including read alignment, achieved by either BWA or CASAVA, variant calling by either GATK or CASAVA, and annotation by Alamut Batch. Variants with a minor allele frequency (MAF) of less than 1% in available were visualized on Alamut Visual software (Interactive Biosoftware) and classified in 5 classes from 1-benign to 5-pathogenic, based on MAF, predicted effect on protein, predicted effect on splicing, databases of pathogenic variations, according to ACMG recommendations.

#### Sanger Dideoxy Terminator Sequencing

Variants of interest were confirmed by Sanger sequencing in probands and segregation analysis was performed in asymptomatic parents and other affected relatives (primers available upon request). Sequencing products were resolved on an ABI3130xl capillary sequencing instrument (Applied Biosystems, Courtaboeuf, France).

#### Bioinformatic Resources

Primers for Sanger sequencing were designed with Primer3 software (http://bioinfo.ut.ee/primer3-0.4.0/). MAF was obtained on ExAC database (http://exac.broadinstitute.org/), EVS (http://evs.gs.washington.edu/EVS/) and 1000 genomes (http://www.1000genomes.org/). Two databases of pathogenic variations, HGMD (http://www.hgmd.cf.ac.uk/ac/index.php) and ClinVar (http://www.ncbi.nlm.nih.gov/clinvar/), were consulted.

## Results

### Genetic analyses

Targeted NGS analysis in foetus IV-3 from family 1 (Foetus 1) identified a homozygous deletion of the first 4 nucleotides at the 5’ end of exon 11 of the *MPDZ* gene (NM_003829.4): c.1291_1294del. Splice prediction software showed no significant alteration of the 3’ splice site of intron 10. Thereby, this out-of-frame deletion is predicted to result in a frameshift leading to a stop codon after 14 aberrant codons: p.(Val431Metfs*14). Sanger sequencing confirmed this homozygous mutation in foetus IV-3 as well as in her affected sib, foetus IV-5 (Foetus 2), with both parents being heterozygous carriers (Family 1; Fig. 1). This mutation was absent from the control population databases ExAC, EVS and 1000 Genomes as well as from the pathogenic variation databases HGMD and ClinVar. Analysis in foetus V-3 from family 2 (Foetus 3) identified a homozygous substitution in position +1 within intron 5, affecting the canonical 5’ donor splice site of intron 5: c.533 + 1G > T. This splice mutation is therefore predicted to result in a frameshift transcript. This variant is very rare in control populations with a single mutated allele from the 120,068 present in ExAC database, in a non-Finish European heterozygous carrier. Sanger sequencing confirmed the variation at a heterozygous state in both parents (Family 2; Fig. 1). Unfortunately no DNA sample was available from affected patient V-1. Analysis in foetus II-3 from family 3 (Foetus 5) identified a homozygous nonsense mutation in exon 17 of *MPDZ*: c.2248C > T; p.(Arg750*). This nonsense variant is also rare with 8 occurrences among the 120,618 ExAC alleles, resulting in a minor allele frequency of 6.63.10^−5^ in this set of control populations. DNA sample was only available for one of the two other affected siblings (II-4, Foetus 6) and for their mother (I-2) in family 3. Sanger sequencing identified this variant in affected sibling II-4 at a homozygous state and in the mother I-2 at a heterozygous state (Family 3; Fig. 1). Exon 5, exon 11 and exon 17 are not alternatively spliced in all three validated RefSeq transcripts (NM_003829; NM_001261406; NM_001261407). These three mutations are expected to introduce a premature stop codon, activating the nonsense-mediated decay (NMD) pathway, and resulting in mRNA degradation and a complete loss of functional protein.

### General autopsy findings

Detailed autopsy findings are presented in table 1. Growth parameters were in accordance with the term in all foetuses but 2 who presented with macrosomia (Foetuses 2 and 3). All displayed a similar, although nonspecific cranio-facial dysmorphism, consisting in severe macrocephaly, hypertelorism and broad nasal ridge, short nose with bulbous tip, prominent philtrum, retrognathism and low set and posteriorly rotated ears (Fig. 2C, 2D). No limb anomalies, in particular adducted thumbs or camptodactyly, were observed. Associated visceral malformations consisted of posterior cleft palate in foetus 2, unilateral pulmonary hypoplasia in foetus 3 and Fallot tetralogy in foetuses 4 and 5.

**Table 1:** General autopsy findings and brain macroscopic characteristics in the five foetuses mutated in the *MPDZ* gene

Case number and sex	TOP	Body weight	Head circumference	Brain weight	External examination	Coronal sections	Third ventricle	Aqueduct of Sylvius	Infratentorial structures
Fœtus 1 female (Family 1)	30 WG	1350 g (50^th^ p)	32 cm (>95^th^ p)	210 g (50^th^ p)	Secondary sulci present Enlarged gyri Opened SF	Biventricular dilatation Thin CC rupture of the septum	Atresia with collapse of the thalami	Atresia	Vermis hypoplasia Diamond-shaped fourth ventricle Normal brainstem
Fœtus 2 female (Family 1)	29 WG	1688 g (>95^th^ p)	35 cm (> > 95^th^ p)	181 g (50^th^ p)	Secondary sulci present Enlarged gyri Opened SF	Biventricular dilatation	Atresia with collapse of the thalami	Atresia	Vermis hypoplasia Normal brainstem
Fœtus 3 male (Family 2)	25 WG	990 g (>95^th^ p)	25 cm (>95^th^ p)	147.5 g (50^th^ p)	Largely opened SF No other fissures	Biventicular and anterior dilatation of the 3^rd^ ventricle	Posterior atresia	Atresia	Asymmetric pyramids and olivary nuclei
Fœtus 4 male (family 3)	29 WG	800 g (10^th^ p)	27 cm (75^th^ p)	NA (autolysis)	Secondary sulci present Enlarged gyri Opened SF	Biventricular dilatation	NA	Atresia	NA
Fœtus 5 male (Family 3)	25 WG	786 g (50^th^ p)	24 cm (>95^th^ p)	NA	Largely opened SF No other fissures	Biventricular dilatation Thin CC	Atresia	Atresia	Normal
Fœtus 6 male (Family 3)	23 WG	482 g (50^th^ p)	21 cm (>95^th^ p)	NA	No fissures	Biventricular dilatation Thin CC	Narrowed 3rd ventricle	Atresia	Normal

*TOP* Termination of the pregnancy, *WG* weeks of gestation, *p* percentile, *SF* Sylvian fissure, *CC* corpus callosum, *NA* not available

### Neuropathology

#### Macroscopy

Detailed brain macroscopic characteristics are presented in table 1. Brain weights were in accordance with the term despite hydrocephalus. On external examination, gyration was concordant with the term but the gyri were broadened and the Sylvian fissure largely opened. Olfactory bulbs and optic chiasm were present in all cases. On supratentorial coronal sections ventricular dilatation was severe, with a considerable thinning of the cerebral mantle and in 2 cases and rupture of the septum. The third ventricle was either absent with a collapse of the thalami, or severely narrowed (Fig. 2e). In case 2, severe ventriculomegaly was responsible for parenchymal loss resulting in a porencephalic-like cavity at the anterior frontal midline (Fig. 2f). On sections passing through the mesencephalon, the aqueduct of Sylvius was identified in none of the cases (Fig. 2g). Some additional features included abnormal shape of the fourth ventricle in a single case (Fig. 2h), and asymmetry of pyramids in case 3. Cerebellar hypoplasia was observed only in family 1.

#### Histology

**Fig. 3:**
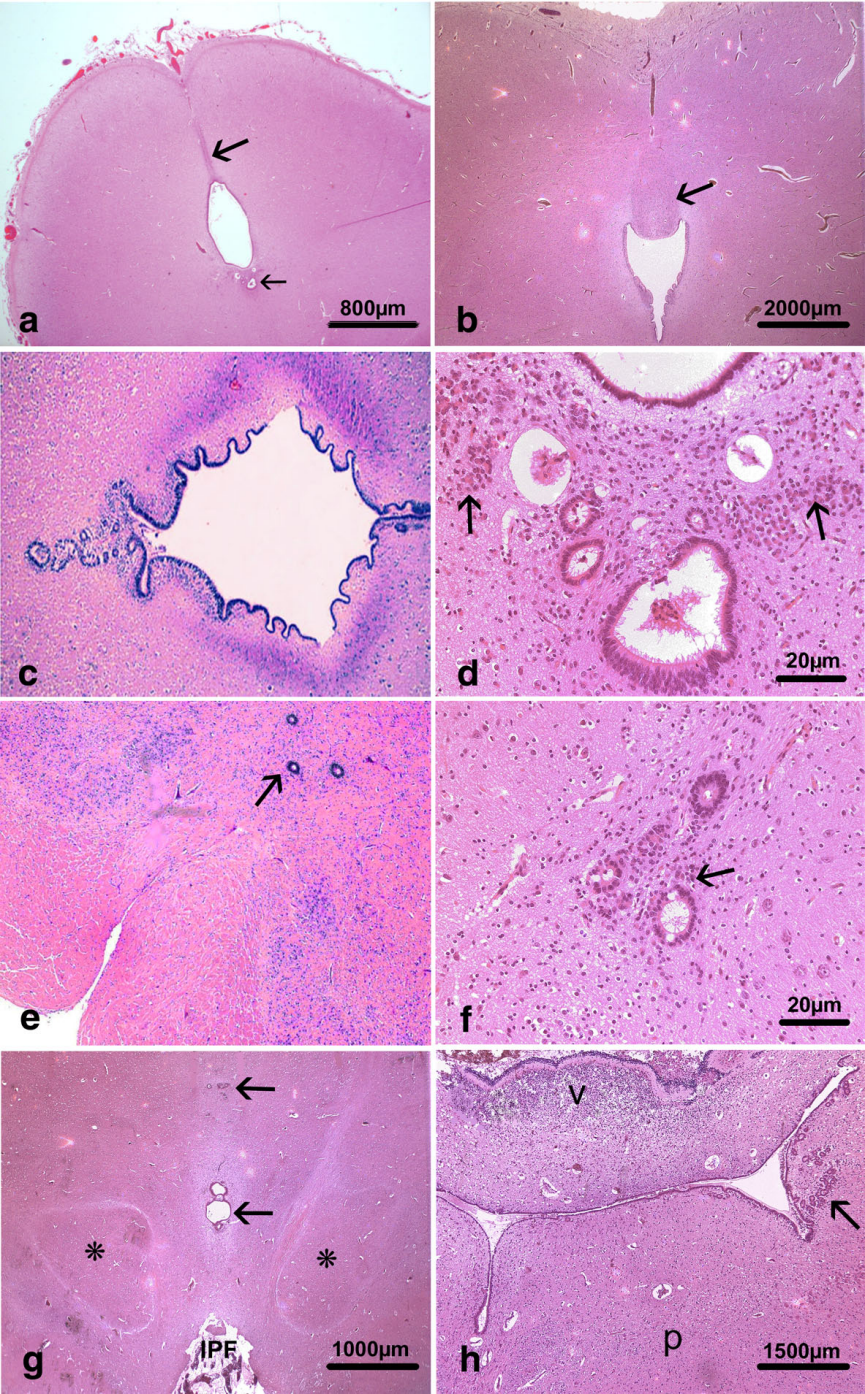
Main histological lesions consisted in hypoplasia of the SCO (*thick arrow*) and colliculi, small aqueduct with rosettes in its inferior part (*thin arrow*) (**a**) compared with an age-matched control whose SCO has a normal size (*arrow*) (**b**) In foetus 4, the patent cerebral aqueduct possessed multiple indentations with rosettes in its dorsal and ventral parts [OM x 100] (**c**) At higher magnification the rosettes were most often lined by ciliated ependyma, and surrounded by small round-shaped cells (*arrows*) (**d**) and in foetus 4 atresia of the ependymal canal was observed at the level of the decussation of the pyramids (*arrow*) [OM x 20] (**e**) The immature cells were either dispersed or clustered (*arrow*) (**f**) and similar lesions observed around third ventricle (*arrows*) at the level of the interpeduncular fossa, between the red nuclei (*asterisks*) (**g**) as well as close to the 4^th^ ventricle ependyma of the fourth ventricle (*arrow*) which was abnormal in shape (**h**)

Histologically, all brains displayed identical lesions, the main being located in the mesencephalon, where the sub-commissural organ (SCO) was hypoplastic compared to control brains and without fusion of the colliculi (Fig. 3a, b). Atresia-forking of the aqueduct of Sylvius was constantly observed, consisting in a patent but narrowed lumen forming multiple indentations (Fig. 3c). The lumen was surrounded by multiple small tubules lined by ependymal ciliated cells forming rosettes, with additional clustered or dispersed cells around the aqueduct, and mostly observed in its ventral part. These round-shaped and small-sized cells possessed a slightly vesicular central nucleus, eosinophilic cytoplasm and well-defined plasma membrane (Fig. 3d). These lesions were associated to multiple foci of ependymal denudation predominating in the dorsal part of the aqueduct where specialized ependyma of the SCO is normally observed. Similar lesions were also noted more caudally in the central canal of the medulla at the level of area postrema, extending to the level of decussation of the pyramids (Fig. 3e, f). Moreover, the same lesions were present ventrally to the third ventricle, where ependymal irregularities were of variable severity (Fig. 3g) and in the lateral parts of the 4^th^ ventricle, with associated deformation of the lumen (Fig. 3h). The internal capsule was normal in volume, shape and direction and callosal fibres were present. At last, no malformative or acquired lesions were observed in any of the different infra- and supratentorial brain structures analyzed.

#### Immunohistochemical studies

**Fig. 4:**
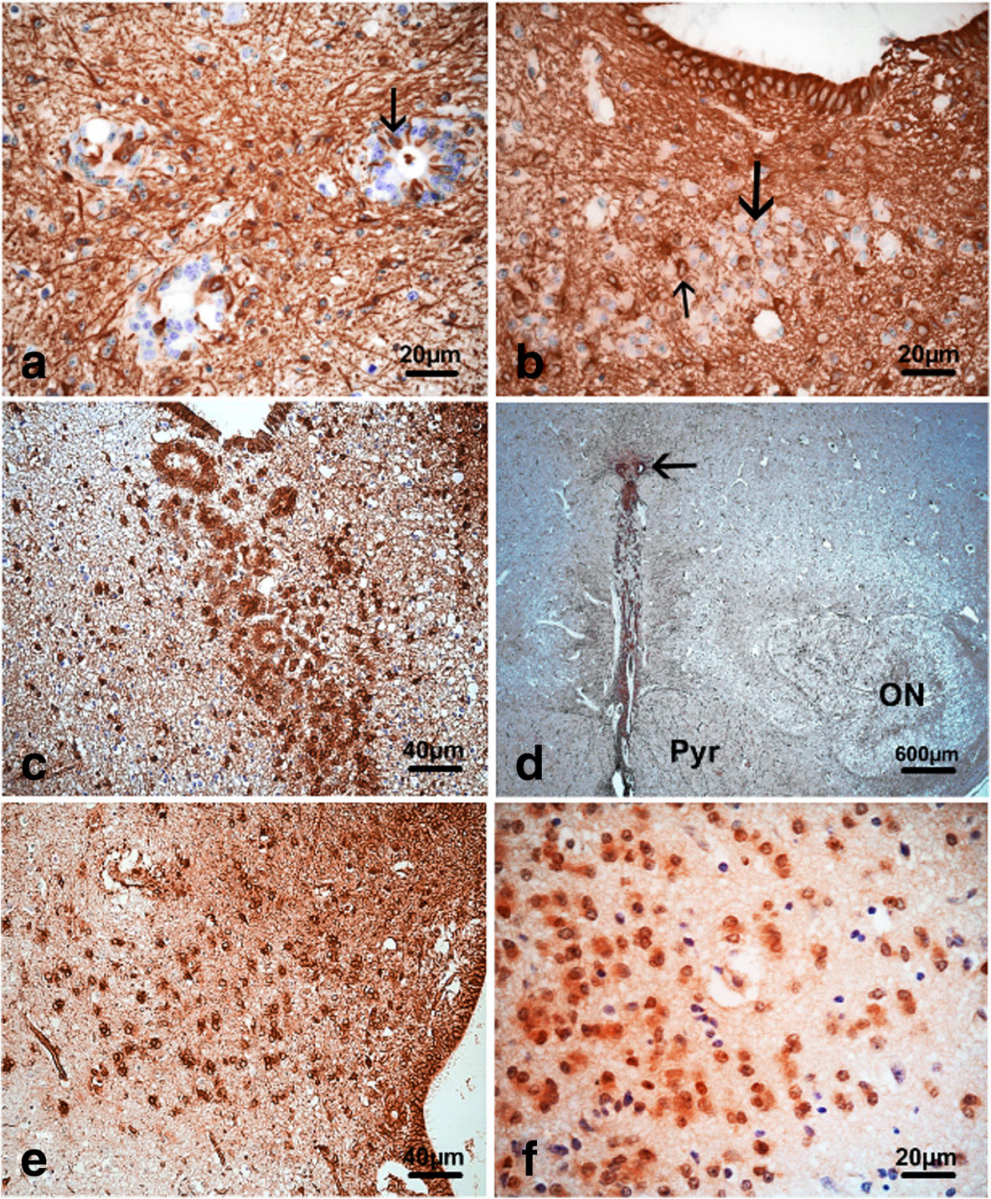
Immunohistochemical findings using GFAP antibody showed only scattered immunoreactive ependymal cells within the rosettes (*thin arrow*) (**a**) along with reactive gliosis *(thin arrow*) but clustered or isolated cells were negative (*thick arrow*) (**b**) rosettes and cells were strongly S100B immunoreactive (**c**) as well as vimentin positive in the ependyma of the central canal of the medulla (*arrow*) (**d**) and surrounding cells were also immunolabeled using nestin and SOX2 antibodies respectively (**e, f**)

GFAP immunostainings revealed a strong immunoreactivity in the ependymal lining of the aqueduct of Sylvius, the fourth ventricle and in the central canal of the medulla. Conversely, this antibody immunolabeled only scarce ependymal cells within the tubules, and clustered or dispersed cells around the ependymal rosettes were negative (Fig. 4a, b). These cells were surrounded by reactive astrocytosis which was also identified around and within in the SCO Ependymal apical plasma membranes and kinocilia were EMA-positive, as in controls of the same age. The pan-cytokeratin marker AE1-AE3 was negative in the patients and controls. S100B protein immunolabeled most of clustered or dispersed cells, as well as cells from the tubules and from the SCO (Fig. 4c), which was also positive for vimentin (Fig. 4d), unlike in controls where vimentin immunoreactivity disappears after 20WG. Notably, the dispersed or clustered cells were negative for vimentin conversely to the fibre network and specialized ependyma of the SCO. In the control brains, CD56 immunolabeled scattered ependymal cells, whereas this antibody was negative in ependymal cells and in dispersed or clustered cells. Nestin was strongly immunoreactive in the SCO similarly to ependymal lining and clustered or isolated cells (Fig. 4e). Moreover, these cells were immunolabeled by SOX2 antibody which immunolabels intermediate progenitors expressed in the inner and outer subventrivcular zones (Fig. 4f).

#### Confocal studies

**Fig. 5:**
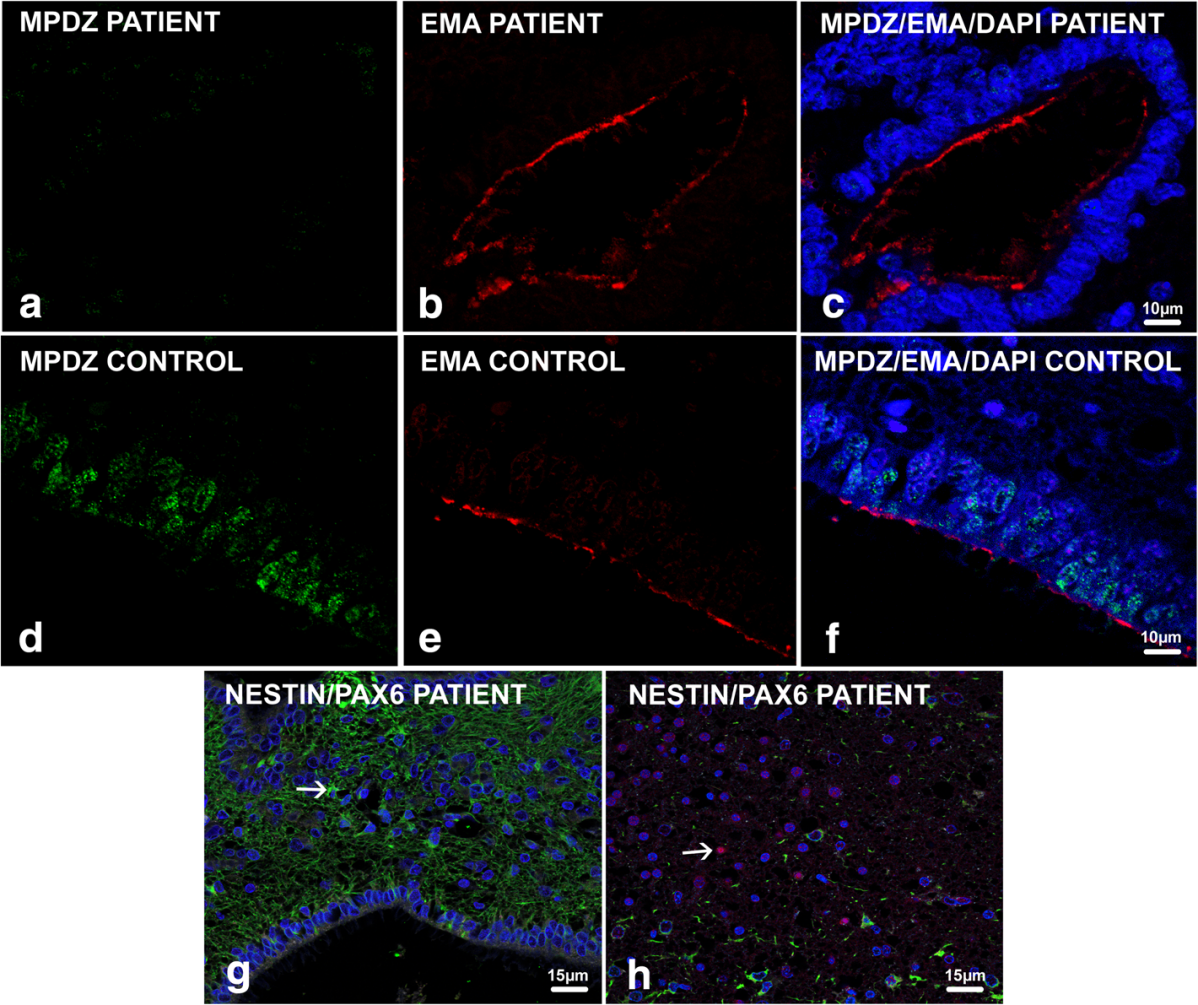
Confocal analyses performed on ependyma sections double stained with anti- MPDZ (*green*) and EMA (*red*) showed a lack of MPDZ positivity in foetus 1 with EMA immunoreactivity at the apex of ependymal lining (**a, b** and **c**) contrary to the control where multiple MPDZ dots (*green*) were strongly expressed at the apical and lateral sides of the ependyma cell membrane and in the underlying cytoplasm (**d, e** and** f**) Immature cells surrounding the aqueduct and rosettes were only immunoreactive for nestin (*arrow*) (**g**) compared to the control case in which the dentate gyrus (used as a positive control) contained multiple stem cells co-expressing nestin (*green cytoplasmic*) and PAX6 (*red nuclear*) (*arrow*) (**h**)

Double EMA/MPDZ immunolabelings revealed absence of MPDZ protein in the patients (Fig. 5a, b and c) compared with an age-matched control where MPDZ protein was observed apically and laterally on the ependymal plasma membrane and within the underlying cytoplasm (Fig. 5d, e and f). Double nestin/PAX6 immunolabellings revealed that the cells and rosettes were negative for the radial glial marker PAX6 (Fig. 5g, h).

## Discussion

The *MPDZ* gene, located on chromosome 9p24-p22, was first involved in severe non-syndromic congenital hydrocephalus by the characterization of a homozygous nonsense variant in exon 6 in two Saudi consanguineous families, c.628C > T; p.(Gln210*), suggesting a founder effect [2]. In the two index case foetuses, US examination showed severe bilateral symmetrical dilatation of the lateral ventricles with dangling of the choroid plexuses, probable callosal dysgenesis or agenesis and inadequate visualization of the cavum septum pellucidum. The third ventricle could not be visualized and there was a notable thinning of the cerebral cortex. Neuropathological examination was not performed and therefore, the morphological characteristics remained unknown. Genetic testing using a gene panel for non-syndromic congenital hydrocephalus led us to identify a second *MPDZ* homozygous pathogenic variant in a foetus born to consanguineous Senegalese parents (Family 1; Fig. 1) who displayed atresia/forking of the aqueduct of Sylvius, suggesting that the specific phenotype of hydrocephalus due to atresia/forking of the aqueduct of Sylvius results from *MPDZ* mutations. We therefore decided to screen the *MPDZ* gene using this gene panel in the sub-group of 21 foetuses with atresia-forking of the aqueduct of Sylvius belonging to 17 families we previously identified [1]. This approach allowed the identification of two other *MPDZ* homozygous pathogenic variations (Families 2 and 3; Fig. 1). All these four pathogenic variants are null variants: 2 nonsense variants, 1 frameshift deletion and 1 splice variant. It is worth noting that all these pathogenic variants were only detected in foetuses born to consanguineous parents, suggesting that the frequency of heterozygous pathogenic variants in the general population is very low. To assess this prevalence, we examined the ExAC database variants in the *MPDZ* gene. Among 2,210 *MPDZ* variants in the 60,706 ExAC control individuals, 80 are canonical splice site, nonsense or frameshift mutations. These 80 inevitably truncating variants have been found in 155 heterozygous individuals, resulting in an estimation of heterozygous carriers of ~1/624, highlighting the rarity of loss-of-function *MPDZ* alleles in the general population. Accordingly, MPDZ constitutes a rare cause of congenital hydrocephalus and should be considered first and foremost in consanguineous families. Nevertheless, the phenotype associated with less severe mutations such as missense or non-canonical splice variants may be different and remains to be described.

In humans, HSAS encompasses approximately 5–15% of congenital hydrocephalus cases with a genetic cause. When excluding X-linked hydrocephalus, the frequency of non-syndromic forms is very low. Empiric recurrence risk rates range from <1 to 4% [32], indicating the rarity of autosomal recessive congenital hydrocephalus. In HSAS, stenosis of the aqueduct of Sylvius is a hallmark of the disease together with hydrocephalus, adducted thumbs, pyramidal tract agenesis/hypoplasia and corpus callosum agenesis/hypoplasia [1, 27]. *AP1S2* gene pathogenic variants (MIM#300629) which cause hydrocephalus with mental retardation are associated with calcifications and iron deposits in the basal ganglia [20].

Contrasting with the rarity of non-syndromic hydrocephalus known disease-causing genes in humans, a plethora of hydrocephalus mouse models have been generated. Many congenital hydrocephalus genes/loci have been recognized, allowing the search for possible molecular and cellular pathophysiological mechanisms. About ten years ago, Zhang et al. [32] proposed a classification into four subgroups. The first consisted in disruption of neural cell membrane proteins. Second, hydrocephalus could be caused by malfunction of ependymal cell cilia and related proteins; third, by malfunction of mesenchymal cells and perturbation of growth factor signalling pathways and fourth by extracellular matrix disruption. From the present work and from several previous studies, another key pathophysiological mechanism should be discussed, consisting in cell membrane junction component alterations, i.e., adherens and gap junctions as well as cell to cell adhesion molecules (especially N-cadherin) which normally join neuroepithelial cells together from embryonic stages, as early as the 4^th^ post-conception week in humans. These alterations have been shown in spontaneous or engineered mutant animal models to be responsible for development of congenital hydrocephalus by disrupting the VZ with subsequent loss of neuroepithelial cells and later ependyma denudation [3, 12, 16, 18].

Tight junctions (TJs) connect adjacent cells so tightly that they constitute a paracellular barrier that prevents the diffusion of solutes, lipids and proteins across the epithelial cell sheets [26]. Therefore, any disruption of TJs and particularly abnormal cell-cell adhesion by PDZ proteins including MPDZ (also named MUPP1 which is strongly expressed in choroid plexuses) most likely alters the distribution of TJs that leads to uncontrolled secretion of CSF and hydrocephalus. Besides, PDZ proteins are modular proteins that act as adaptors by selective interactions of their PDZ domains to other protein modules [4]. They are localized to specialized submembranous sites including synaptic, tight, gap and neuromuscular junctions. MUPP1 which is concentrated at TJs contains 13 PDZ domains, and constitutes a scaffold for several other tight junction components such as claudins, occludin (Ocln), JAMs, angiomotin (Amot), Amot-like 1 and 2 [9, 23, 25]. The neuropathology in humans has been described only in a minority of mutations in other tight junction component genes and differs from the phenotype observed in case of *MPDZ* mutations. Recessive mutations in the *OCLN* gene (MIM#251290) also named pseudo-TORCH syndrome, cause microcephaly with band–like calcifications, simplified gyral pattern and polymicrogyria. OCLN is mainly expressed in the endothelium, pericytes and surrounding astrocytes [15], and the defective protein leads to destructive lesions. Among the claudin family, only *CLAUDIN1* mutations have been described (MIM#603718) and cause ichthyosis, vacuolated leukocytes and alopecia but without brain lesions. Besides, human brain lesions remain unknown in case of *JAM1* and *JAM4* mutations (MIM#610638). JAM1 also interacts with another PDZ-containing domain, Afadin which is localized at the ependymal zonulae adherens and TJs of the third ventricle and of the aqueduct of Sylvius and whose genetic deletion induces hydrocephalus with disappearance of ependyma in the mouse midbrain and obliteration of the third ventricle [31]. But again, no human pathology has been reported until now. Although it has been demonstrated that Amot colocalizes with occludin in mice, no human pathology has been associated with mutations in the *AMOT* gene so far. Several individuals from a single large family with autosomal recessive *JAM3* mutations [13] presented all bilateral cataracts and some of them had hepatomegaly and thrombocytopenia. MRI displayed multifocal intra-parenchymal haemorrhages in the white matter and basal ganglia with secondary ventricular dilatation owing to major JAM3 expression in the vascular endothelium.

MUPP1 expression has been documented using immunocytochemistry in adult mouse brain, with highest expression on the apical surface of the choroid plexuses, strong expression in the hippocampus, amygdala and pyriform cortex, in Layer II of most neocortical areas as well as in all layers of the cerebellar cortex and in several brainstem nuclei [9, 22], but its expression in human brain is unknown. In our control case, MUPP1 was strongly expressed in the aqueduct ependyma, conversely to the patients in whom a total lack of expression was observed. The ependyma derives from radial glial cells (RGC) which are highly polarized cells joined by zonulae adherens, gap and tight junctions and constitutes a barrier between parenchyma and ventricles from embryonic stage. Consequently, early tight junction alterations lead to abnormal organization of ependyma with rosette formation and narrowed ventricular lumens [10]. In our foetuses, they were observed at different locations with aberrantly located cells in the surrounding parenchyma through loss of cohesion. These cells had the characteristic immunophenotype of RGC-intermediate progenitors (SOX2 positivity; PAX6 negativity) and of non-differentiated ependyma (nestin positivity; vimentin, GFAP and CD56 negativity). At last, we observed that the SCO was hypoplastic, probably due to early denudation of the specialized ependyma of the aqueduct leading to SCO ependyma malfunction, since SCO secretions support the integrity of ependymal cells in this region and prevent the closure of the cerebral aqueduct [5, 11, 29].

## Conclusions

We describe three novel homozygous null mutations in the *MPDZ* gene in foetuses whose post-mortem examination has revealed a homogeneous phenotype characterized by multifocal ependymal malformations along the aqueduct of Sylvius, the third and fourth ventricles as well as the central canal of the medulla, with immature cell accumulation in the vicinity of ependymal lining, highlighting for the first time that primary ependymal malformation of the ventricular system is genetically determined in humans. Therefore, *MPDZ* sequencing should be performed when neuropathological examination reveals multiple ependymal malformations of the aqueduct of Sylvius, of the third and fourth ventricles and of the central canal of the medulla.

## Abbreviations

IPF: Interpeduncular fossa
OM: Original magnification
V: Vermis
P: Pons
